# Supplementary material for: Phase management in single-crystalline vanadium dioxide beams
Source: Nat Commun. 2021 Jul 9;12:4214. doi: 10.1038/s41467-021-24527-5 (PMC8270972; doi:10.1038/s41467-021-24527-5)
Supplement: Supplementary file 1 — Supplementary Information [file 41467_2021_24527_MOESM1_ESM.pdf]

Supplementary Information for

Phase Management in Single-Crystalline Vanadium Dioxide Beams

Run Shi[1,2], Yong Chen[1,2], Xiangbin Cai[2], Qing Lian[1], Zhuoqiong Zhang[1], Nan Shen[1], Abbas Amini[3], Ning Wang[2] and Chun Cheng[1]*

[1] Department of Materials Science and Engineering, Southern University of Science and Technology, Shenzhen 518055, P. R. China

[2] Department of Physics and Center for Quantum Materials, Hong Kong University of Science and Technology, Hong Kong, P. R. China

[3] Center for Infrastructure Engineering, Western Sydney University, Kingswood, NSW 2751, Australia

* E-mail: chengc@sustech.edu.cn

Supplementary Figures

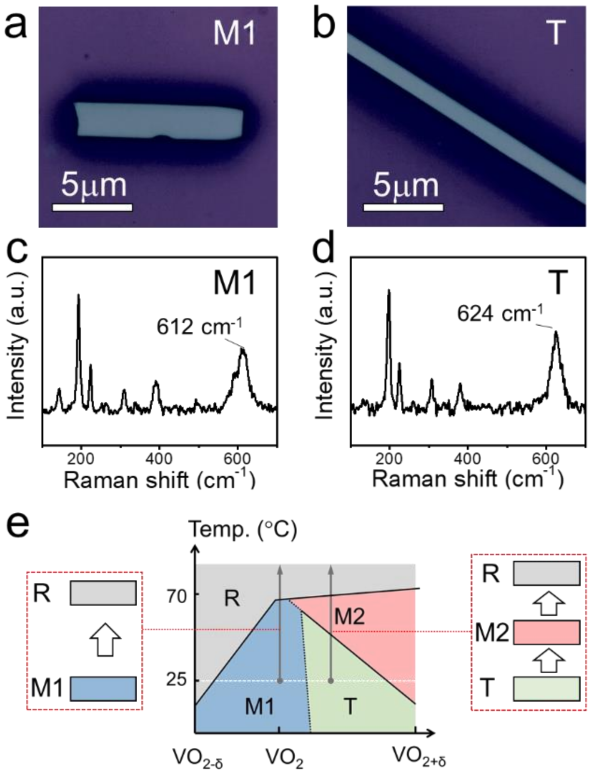

Supplementary Figure 1. Comparisons between M1 and T phase VO_2. Optical images of (**a**) M1 phase, and (**b**) T phase VO_2 beam captured under similar conditions. Raman spectra of selected (**c**) M1 phase and (**d**) T phase VO_2 beam. (**e**) Different phase transition routes of M1 phase VO_2 and T phase VO_2.

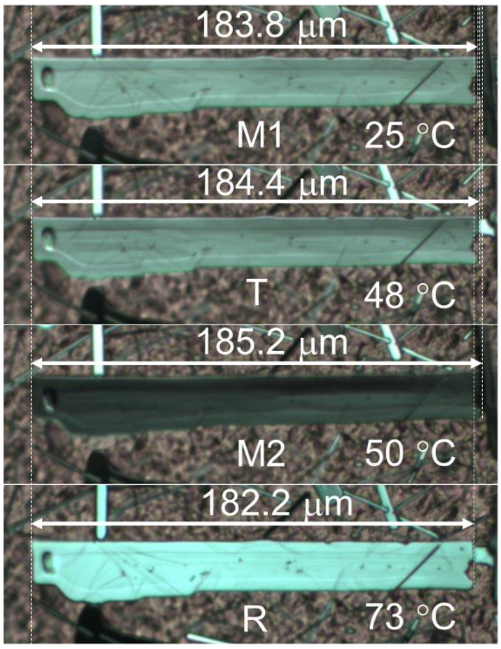

Supplementary Figure 2. Temperature-dependent optical images of VO_2 beam undergoing M1→T→M2→R transition route.

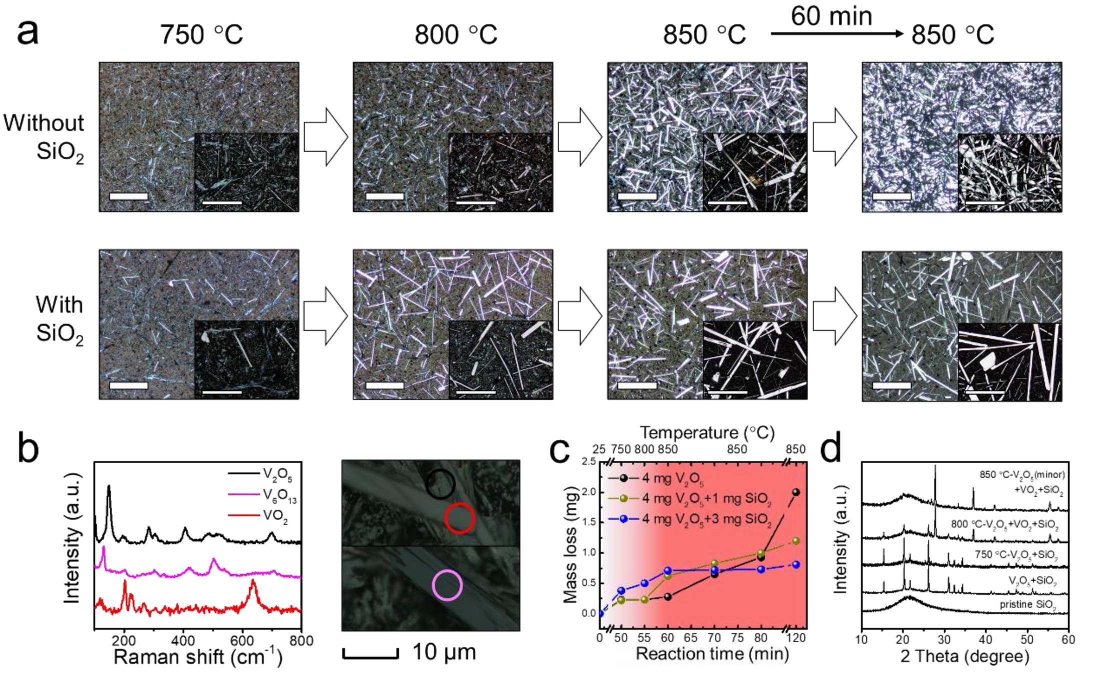

Supplementary Figure 3. Influence of SiO_2 inhibitor on the CVD growth of VO_2 beams. (**a**) Optical images of VO_2 beams prepared under different conditions (scale bar, 200 μm). Insets show the magnified view of the optical images (scale bar, 100 μm). (**b**) Raman spectra of the products prepared at 800 °C with different colors and morphologies. The colored circles in the right optical images show the positions of measurements, corresponding to the Raman data with the same color. (**c**) Plots of mass loss of the oxide mixture during the CVD reaction with different amounts of SiO_2 added to the system, where the colored background shows the change of reaction temperature. (**d**) XRD patterns of pristine SiO_2, the oxide mixture of SiO_2 and V_2O_5 before reaction, and the oxide residues after the reactions at different temperature.

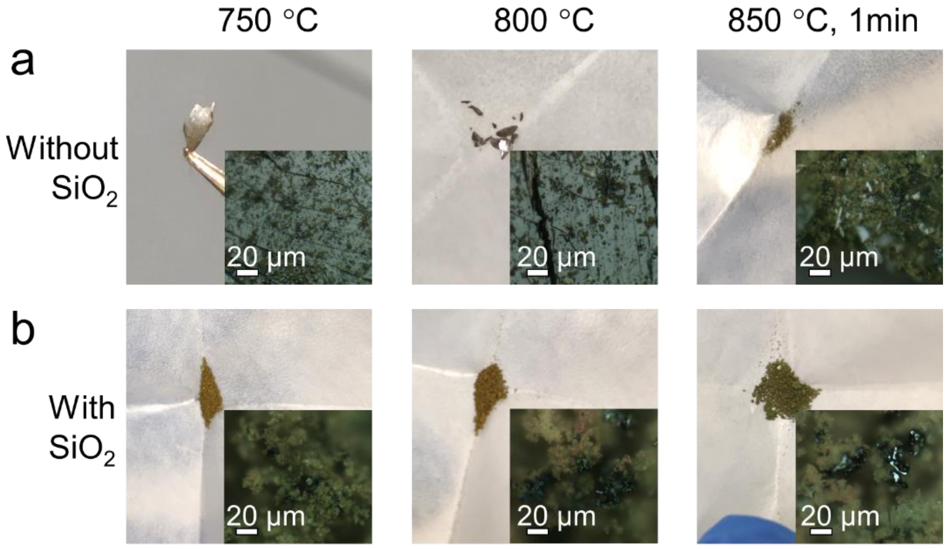

Supplementary Figure 4. Optical images of reaction residues. Optical images of reaction residues of the oxide precursors during the CVD reaction in the (**a**) absence and (**b**) presence of SiO_2. The insets are the magnified view of the oxide residues.

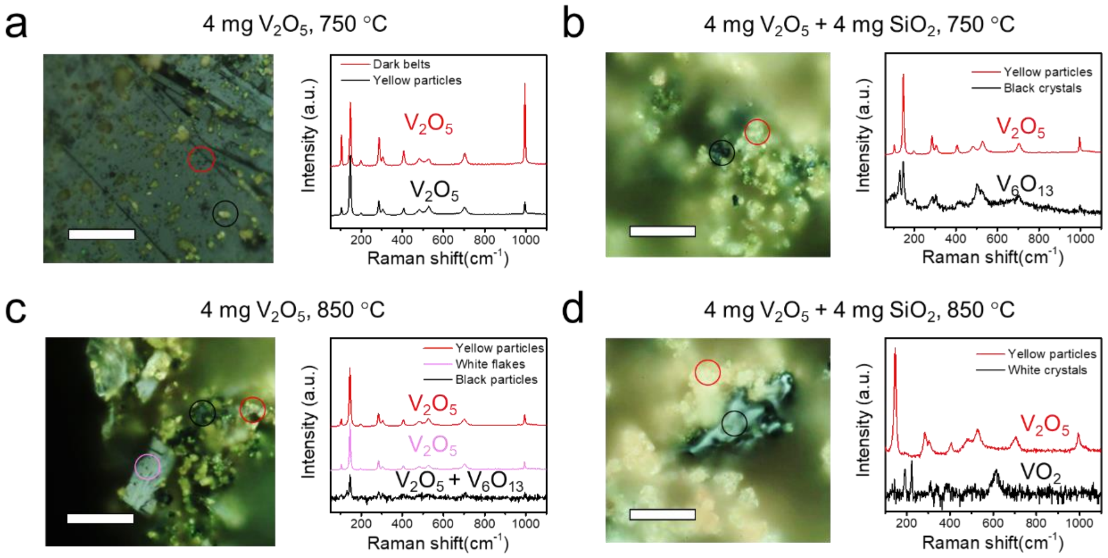

Supplementary Figure 5. Raman spectroscopy of reaction residues. Optical images (scale bars are 20 μm) and Raman spectra of oxide residues under: (**a**) 4 mg V_2O_5 at 750 °C; (**b**) 4 mg V_2O_5 mixed with 4 mg SiO_2 at 750 °C; (**c**) 4 mg V_2O_5 at 850 °C; and (**d**) 4 mg V_2O_5 mixed with 4 mg SiO_2 at 850 °C. The colored circles show the positions of measurements, linked to the Raman data with the same colors.

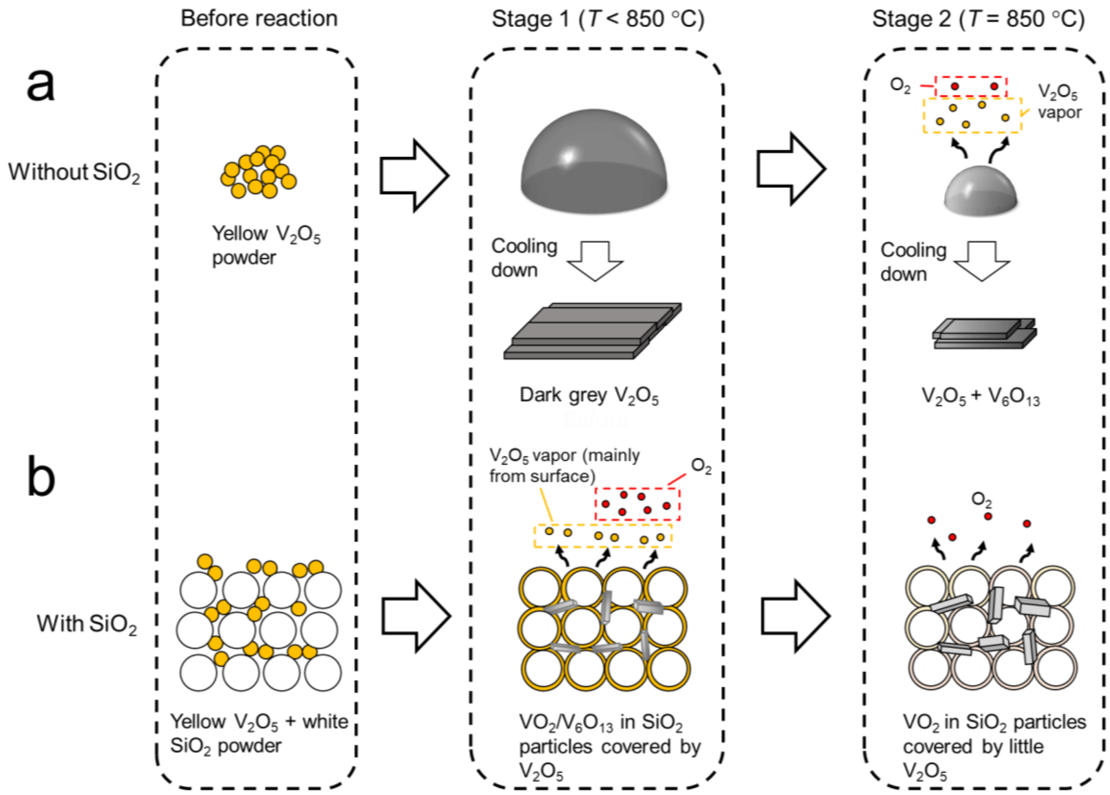

Supplementary Figure 6. Mechanism for the SiO_2 inhibitor-assisted CVD reactions. Schematics for the status of oxide precursors during the CVD reaction in the (**a**) absence and (**b**) presence of SiO_2.

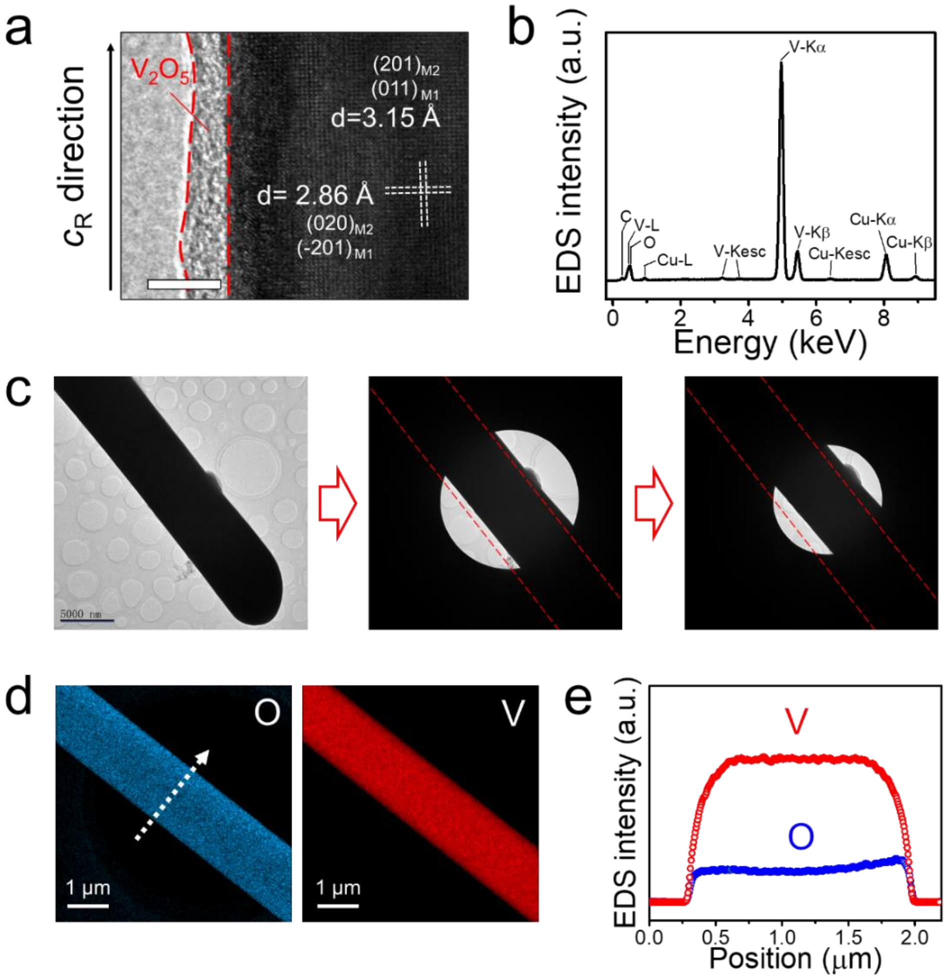

Supplementary Figure 7. Characterizing nonstoichiometric VO_2 beams. (**a**) HRTEM image of as-grown non-stoichiometric VO_2 beam (scale bar is 5 nm). (**b**) EDS spectrum of VO_2 beam. (**c**) TEM images of the beam with the increasing dose of incident electron beam. (**d**) EDS mapping of O and V along a washed T-M2 type SCVA. The white arrow shows the scan line along the lateral direction. (**e**) Element distribution of V and O across the lateral direction.

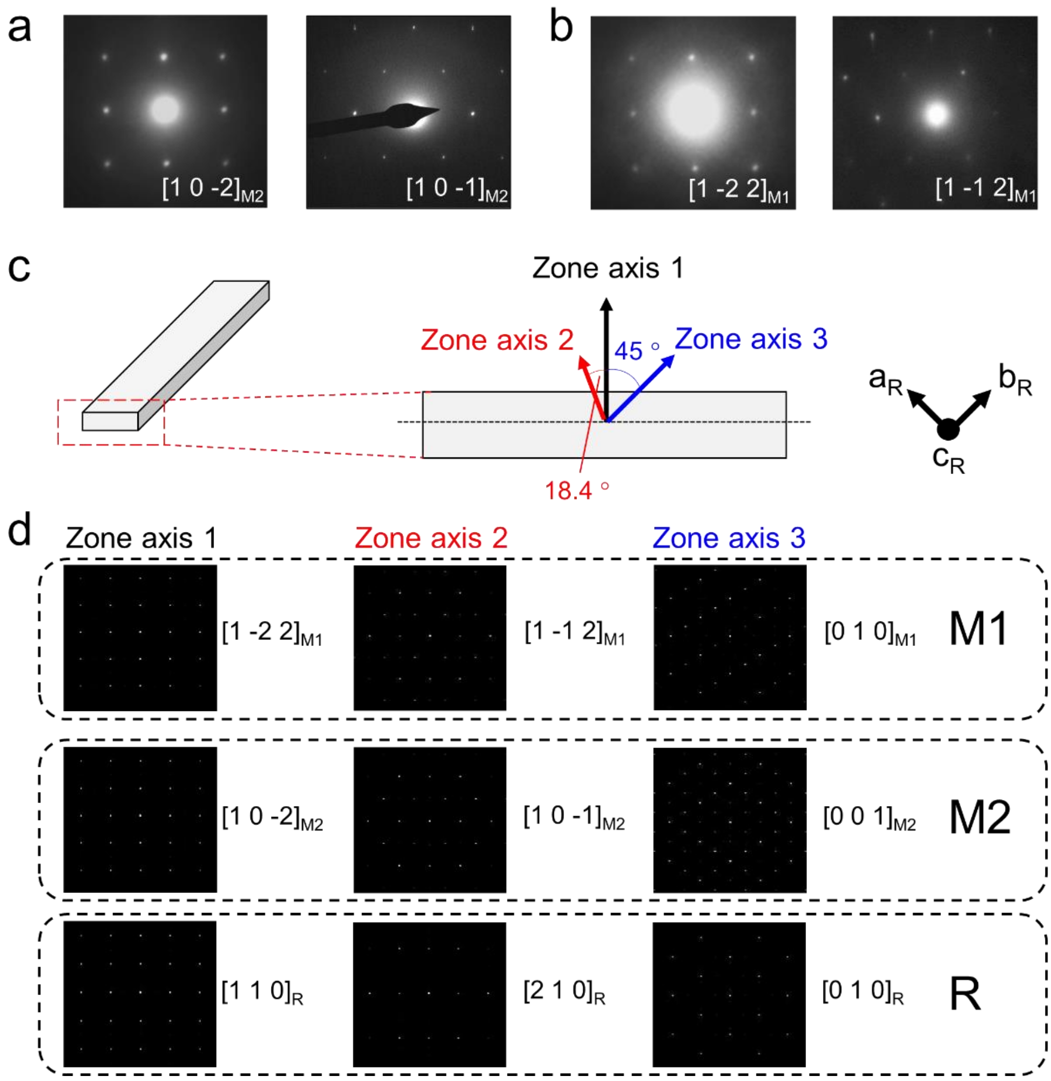

Supplementary Figure 8. SAED of single-crystalline VO_2 beams. (a) Experimental SAED patterns of a M2 phase VO_2 beam at the zone axis $[1\ 0\ -2]_{M2}$ and zone axis $[1\ 0\ -1]_{M2}$. (b) Experimental SAED patterns of a M1 phase VO_2 beam at the zone axis $[1\ -2\ 2]_{M1}$ and zone axis $[1\ -1\ 2]_{M1}$. (c) Schematic of as-grown VO_2 beams with three primary zone axes 1-3 normal to the axial direction (c_R) of VO_2 beam. (d) First-principle simulated SAED patterns of M1, M2 and R phase VO_2 single crystals at zone axes 1-3.

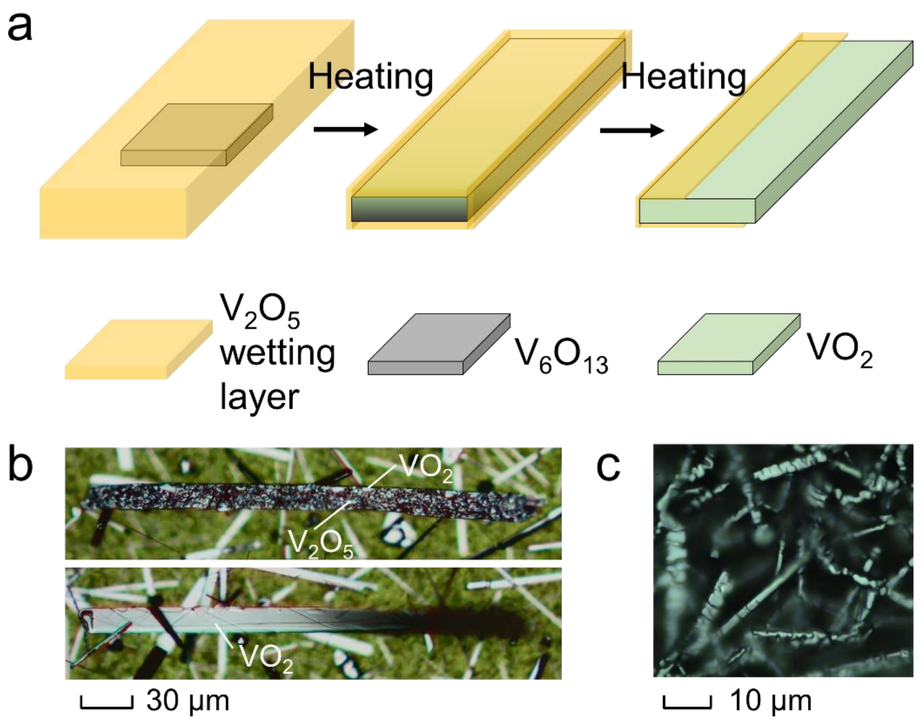

Supplementary Figure 9. Formation of oxygen gradient in VO_2 beams. (**a**) Schematic diagram of wetting layer-assisted CVD growth of VO_2 beams with limited V supply. (**b**) Optical images of two sides of a VO_2 beam partially covered by a V_2O_5 layer. (**c**) Optical image of VO_2 beams reacted with tungsten oxide vapor (W vapor).

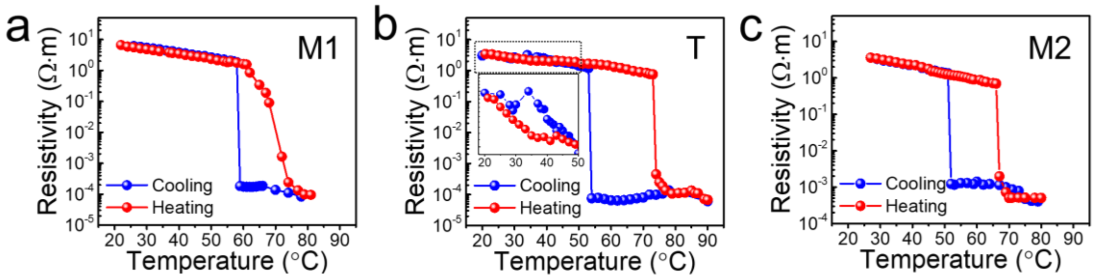

Supplementary Figure 10. Electrical measurements of VO_2 beams. Temperature-dependent resistivity plots of room-temperature-stable (**a**) M1 phase, (**b**) T phase, and (**c**) M2 phase single VO_2 beam. The inset of (**b**) shows the magnified view of resistivity-temperature curves framed by the dashed rectangle.

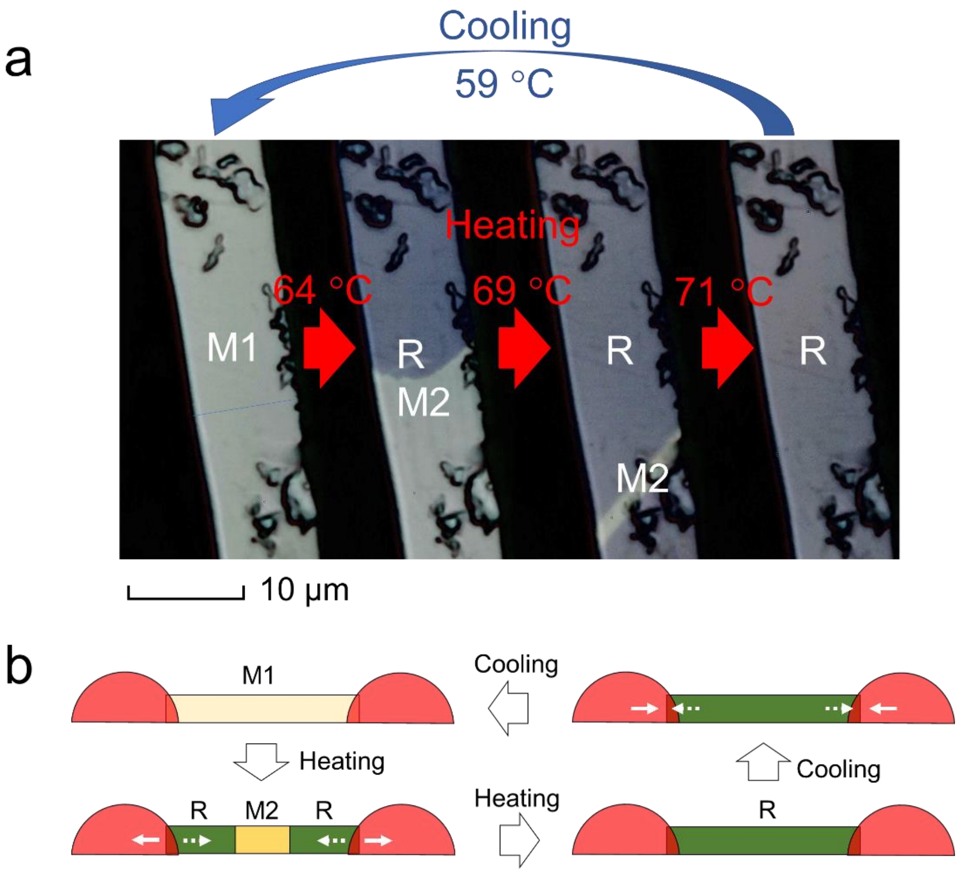

Supplementary Figure 11. Influence of external stress on the electrical measurements of VO_2 beams. Temperature-dependent (**a**) optical images and (**b**) schematic images of the clamped M1 phase VO_2 beam with electrodes (conductive silver epoxy). The dash arrows show the preferred deformation direction of the beam upon the change in temperature, and the solid arrows show the direction of stress applied on the beam by electrodes.

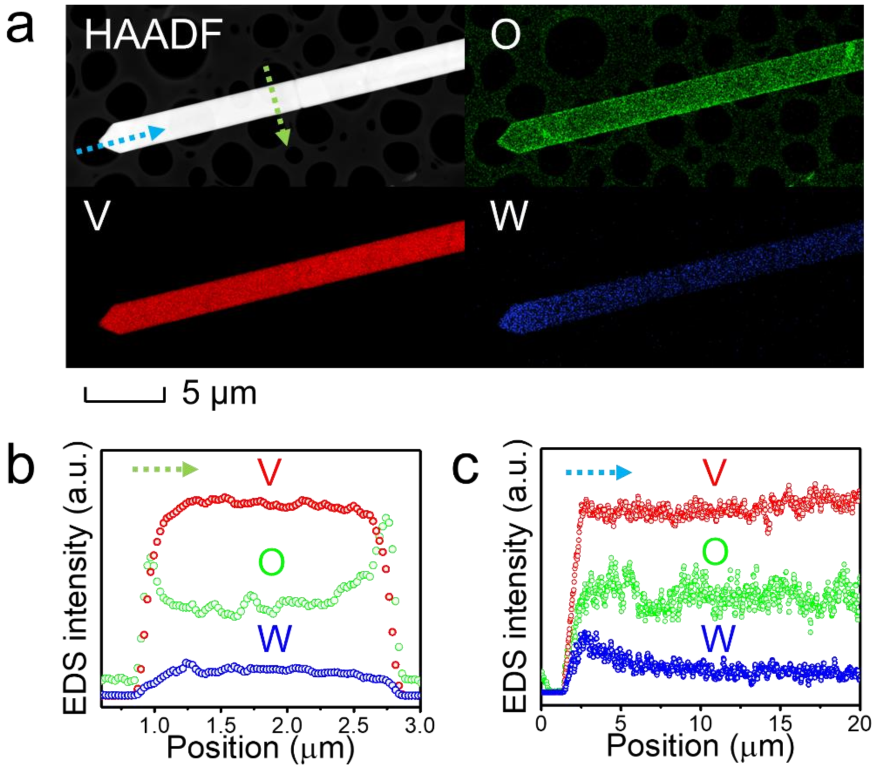

Supplementary Figure 12. Characterizing W-doped VO_2 beams. (**a**) HAADF image with the EDS mapping of V, O, and W along a W-doped M2-R type SCVA. The green arrow and blue arrow show the scan lines along the lateral direction and the axial direction, respectively. Element distribution of V, O, and W across (**b**) the lateral direction and (**c**) axial direction of the beam.

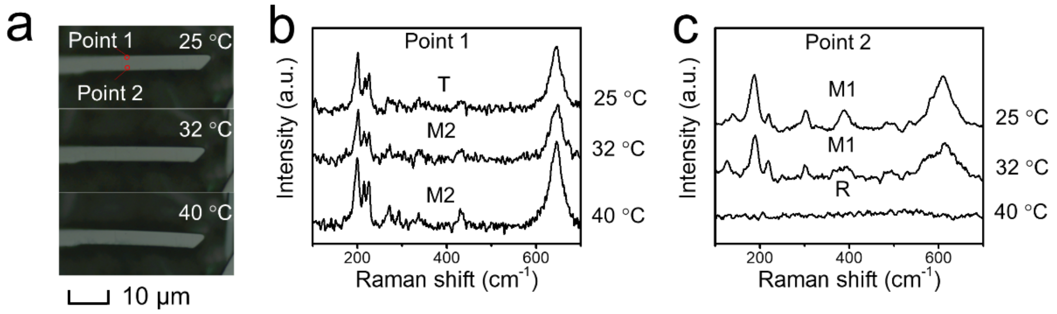

Supplementary Figure 13. Raman spectroscopy of M2-R SCVA. (**a**) Temperature-dependent optical images of a M2-R type SCVA, and (**b-c**) the corresponding Raman spectra of the both sides of SCVA upon heating.

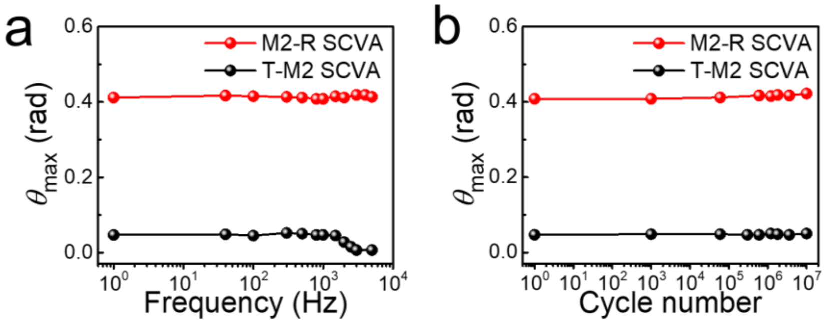

Supplementary Figure 14. Work speed and stability test of SCVAs. (**a**) θ_{max} of oscillating M2-R type SCVA and T-M2 type SCVA driven by a laser pulse with varying pulse frequencies in air. (**b**) θ_{max} versus cycle number of the SCVAs driven by a laser pulse at 1000 Hz.

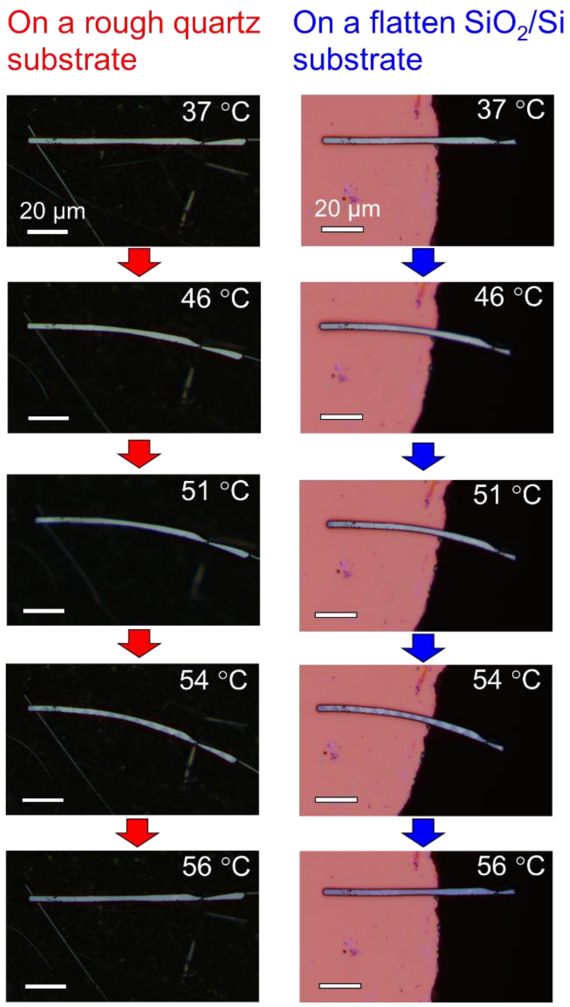

Supplementary Figure 15. Influence of substrate on the phase transition properties of VO_2 beams. Temperature-dependent optical images of (**a**) a self-bending W-doped VO_2 beam on a rough quartz substrate, and (**b**) its images after being transferred to the edge of a smooth SiO_2/Si substrate.

Supplementary Tables

Supplementary Table 1. Latent heat of different VO_2 phase transitions

Phase transition	Latent heat, *H* (cal/mol)
M1-R[1]	~1030
M2-R[2]	~710
T-M2[1]	27-300

Supplementary Discussion

1. Distinguishing M1 phase and T phase in VO_2

As discussed in the main text, it is hard to distinguish M1 phase and T phase in VO_2 via optical microscopy shown in **Supplementary Fig. 1a & 1b**. In addition to the results from Raman spectroscopy, the lattice structure of VO_2 can be distinguished according to its phase transition route. As shown in **Supplementary Fig. 1e**, the pristine M1 phase VO_2 undergoes an abrupt M1→R transition upon heating. By contrast, the formation of M2 phase during the transition indicates that the VO_2 sample is in T phase at room temperature.

However, this method can only identify the T phase with a relatively high oxygen content, because M1 phase may still retain in the slightly oxidized VO_2 at room temperature but there is a complex M1→T→M2→R transition route as shown in **Supplementary Fig. 2**. The change in the length of beam upon heating verifies the continuous transition of M1→T with an ultimate axial strain of ~0.33%. While the calculated strain of 0.76% during the M1→M2 transition matches well with the theoretical value.

2. Influence of SiO_2 on CVD growth of VO_2 beams

V_2O_5 is usually chosen as the vanadium source in the chemical vapor deposition (CVD) growth of VO_2 beams due to it relatively low melting point[3], while it evaporates fast which is hard to control. Researchers have long struggled to find an effective way to control the supply of gaseous precursors, especially the vapor generated by heating solid sources. Recently, we developed the oxide inhibitor (OI)-assisted growth of two-dimensional MoS_2 monolayers[4], where the Mo vapor (MoO_3) release could be finely modulated by inert oxide inhibitors. It was discovered that the thicker OI layer led to a longer diffusion path of Mo vapor through the oxide mixture, and thus a larger portion of Mo source was trapped within the inhibitor layer. Inspired by this work, we

developed a similar OI strategy to control the release of V_2O_5 vapor in the CVD growth of one-dimensional VO_2 beams and achieved a good control of the V vapor supply as well as a continuous control of oxygen partial pressure in the reaction system.

Below, we discuss the working mechanism of SiO_2 inhibitor from physical and chemical points of view:

Firstly, SiO_2 functions as a physical trapping material to inhibit the fast release of V_2O_5 vapor. We comprehensively studied the reaction residues by optical microscopy, XRD, and Raman etc., as shown in **Supplementary Figs. 3-5**. After the CVD reactions, the color of SiO_2 changes from white to yellow or orange (the color of V_2O_5), as shown in **Supplementary Fig. 4b**. This suggests the considerable amount of V_2O_5 is attached to the surface of SiO_2; this is verified by Raman spectra (**Supplementary Fig. 5**). Moreover, XRD and Raman studies of reactant residues show that there are no additional compounds other than the reactants and typical products, including V_2O_5, V_6O_{13}, VO_2, and SiO_2 (**Supplementary Fig. 3d** and **Fig. 5**), suggesting that reaction inert of SiO_2 in the present reaction conditions. We conclude that SiO_2 works as an effective physical trapping material to inhibit the fast release of V_2O_5 vapor.

Secondly, SiO_2 promotes the conversion of V_2O_5 to VO_2, modulating the release of V_2O_5 vapor and oxygen partial pressure. Even though SiO_2 does not directly participate in the reaction, it plays an essential role in modulating the CVD reaction process. We studied the mass loss of reactants *vs*. deposition temperature and time at various addition amounts of SiO_2 as shown in **Supplementary Fig. 3c**, and characterized the corresponding reaction residues. It is not expected to see at $T < 850$ °C, the V_2O_5 evaporation (indicated by mass loss) in the absence of SiO_2 is slower than that in the presence of SiO_2 as shown in **Supplementary Fig. 3c**. This result can be attributed to the unique properties of V_2O_5. V_2O_5 powder tends to melt and merge into a single mass that has a relatively low evaporation rate at $T < 850$ °C; in this condition, the evaporation of V_2O_5 is self-limited. Once the temperature increases to 850 °C, the evaporation of V_2O_5 is accelerated. The optical images in Figure R6a verify the above deduction, and show the obtained residues in the absence of SiO_2 at $T < 850$ °C look

like glassy crystals while they change to powder-like substance at T = 850 °C. In addition, Raman spectra (**Supplementary Fig. 5**) indicate that the main component of the reaction residues at T < 850 °C is V_2O_5 and, at T = 850 °C, they are V_2O_5 with only a small amount of V_6O_{13}. In fact, most of the vapor in the reaction atmosphere should be in the form of V_2O_5 but few O_2 is released from the precursor due to its limited reduction as shown in **Supplementary Fig. 6a**.

By contrast, the addition of SiO_2 breaks the self-limitation of V_2O_5 evaporation phenomenon by dispersing V_2O_5 powder; dispersed V_2O_5 powder has a larger exposed surface and its evaporation is enhanced by SiO_2 at T < 850 °C (**Supplementary Fig. 3c**). Moreover, from the Raman spectra in **Supplementary Fig. 5** and XRD analysis in **Supplementary Fig. 3d**, it is concluded that a large proportion of V_2O_5 source is trapped and reduced to VO_2 during the reaction. It is then accompanied with a large amount of O_2 released to the reaction atmosphere that results in a relatively high oxygen partial pressure as shown in **Supplementary Fig. 6b**. Therefore, the mass loss at T = 850 °C should be very limited considering the low evaporation rate of VO_2 and physical inhibition of SiO_2, which is consistent with the result of **Supplementary Fig. 3c**. This claim can also be supported by the fact that there is no increase or growth of VO_2 beams at T = 850 °C in the SiO_2-assisted CVD reactions as shown in **Supplementary Fig. 3a**. As the reduction of remaining V_2O_5 is ongoing at T = 850 °C, the partial oxygen pressure can be maintained at a relatively high level for the effective stoichiometry engineering of as-grown VO_2 beams.

The optical images in **Supplementary Fig. 3a** further verifies the reaction process from the different points of view. Obviously, at T < 850 °C, the beams in the presence of SiO_2 grow faster than those in the absence of SiO_2. After the reaction at 850 °C for 20 min, upon the increase of SiO_2 dosage, the nucleation density of VO_2 beams is greatly decreased. This is corresponded to the inhibited V_2O_5 evaporation in the presence of SiO_2 and the promoted evaporation with no SiO_2 at T = 850 °C.

In summary, the inhibiting process of SiO_2 in the CVD growth of VO_2 beams is a coupled result of physical inhibition and chemical inhibition. SiO_2 initially promotes

the evaporation at T < 850 °C and then prohibits the evaporation at T = 850 °C, accompanied with the increase in the oxygen partial pressure.

3. Structural and compositional analyses of as-grown VO_2 beams

High-resolution transmission microscopy (HRTEM) and energy-dispersive X-ray spectroscopy (EDS) are used to further characterize the lattice and composition information of as-prepared VO_2 beams. The HRTEM image of an as-grown nonstoichiometric beam in **Supplementary Fig. 7a** has two sets of lattice fringes with an intersecting angle of ~90°, which can be indexed to (201) planes and (020) planes of M2 phase or (011) planes and (-201) planes of M1 phase. In contrast to d $(011)_{M1}$ = 3.2 Å, the measured spacing of 3.15 Å is closer to d $(201)_{M2}$ =3.17 Å.[5,6] Furthermore, the axial direction of the VO_2 beam is confirmed to be in c_R direction, the same as most of the reported single-crystalline VO_2 beams. The EDS spectrum in **Supplementary Fig. 7b** shows that there is no doping element in the beam (the peaks of Cu come from the TEM grid), giving solid evidence to the previous claim that the excessive oxygen accounts for the stabilization of M2/T phase VO_2 at room temperature, excluding the disturbance of the external stress or doping.

Supplementary Fig. 7c gives the TEM images of the self-bending T-M2 SCVA in **Figure 3d** activated by the electron beam. It is noted that there are more oxygen signals at its two sides, which could be attributed to the amorphous V_2O_5 layer as shown in the HRTEM image of **Supplementary Fig. 7a**. To eliminate the surface oxidation or attached V_2O_5 layers, we wash as-grown VO_2 beams by 2M Na_2CO_3 aqueous solution for 10 h[7], and analyze the element distribution of a washed self-bending VO_2 beam. It is noted that the strong O signals at the two sides rather disappear as shown in **Supplementary Fig. 7d** and **7e**.

Selective area electron diffraction (SAED) is a powerful method to precisely characterize the crystal structure of nanomaterials, which should be an ideal method to verify the laterally asymmetric phase structure of self-bending VO_2 beams. However,

SAED requires that the thickness of the samples should be smaller than ~200 nm to allow electron beam pass through for the formation of measurable diffraction pattern, which more or less limits its application range. However, we found it hard to work on the as-grown beams in our work, especially the VO_2 beams with lateral oxygen gradient. It is noted that the difficulty should come from many aspects, including the big size of the interested samples, very close lattice structures of different VO_2 phases, the possible perturbations from the structural distortion or the overlapping of several domains with different phases and orientations at the bending part of the studied beam, and the inevitable heating effect of electron beam. We would like to address these failure and experience when trying SAED on our samples as follow:

1) The relatively large thickness of the beams with oxygen gradient: According to Supplementary Movie 1 and Supplementary Movie 2, it is noted that the radical oxygen gradient usually exists in the VO_2 beams with a large radial size (usually > 1 μm). We have imaged the self-bending beams in **Supplementary Fig. 7c** via SAED. According to the bright-field TEM images, it is observed that the thickness of this beam is too thick to allow either the light or electron transmission, resulting in its completely black contrast in the TEM image. As a result, we failed to obtain the SAED images of the VO_2 beams with an evident radical oxygen gradient.

2) Quite similar lattice structures of VO_2 phases: We studied a thin VO_2 beam with the room-temperature-stable M2 structure via SAED; the SAED patterns at two usual zone axes of $[1\ 0\ -2]_{M2}$ and $[1\ 0\ -1]_{M2}$ are shown in **Supplementary Fig. 8a** for reference. It is noted that the SAED patterns of M2 phase are completely overlapped with those of the typical M1 phase (**Supplementary Fig. 8b**). This result can be understood by considering the similar crystal structures of these phases with quite close periods (d_R=5.700, d_{M1}=5.755, d_T=5.755~5.797 and d_{M2}=5.797, all in Å). All the period differences are smaller than 0.1 Å, and the difference between M2 and T phase should be < 0.05 Å, which seems to be hard to distinguish by SAED.

Different forbidden diffraction points in the SAED patterns may be used to identify different VO_2 phases for the thick VO_2 beams. We thus provide the simulated SAED

patterns along three usual zone axes as shown in **Supplementary Fig. 8c** and **8d**. It is discovered that one cannot distinguish the VO_2 phases at zone axis 1. R phase can be distinguished at the zone axis 2, and all these phases can be identified at the zone axis 3. However, the as-grown VO_2 beams, including those with the oxygen gradient, usually have a rectangular cross-section and always is positioned with the longitudinal edge perpendicular to the zone axis 1, as reported in a previous publication[8]. In real experiments, it is very hard for the TEM operators to tilt the beam by 45° towards the zone axis 3 from its preferential state that is normal to the zone axis 1, because the tilt range of TEM sample stage is usually limited to ±30°. In conclusion, it is difficult to apply SAED to identify the local crystal structure of VO_2 beams, because the tiny difference of the crystal lattices of VO_2 phases cannot produce measurable difference in the SAED patterns at the preferential zone axis of as-grown VO_2 beams.

In summary, we think that SAED may not be a proper method to characterize the unique phases of VO_2. The clarification on the weakness of SAED in our case above may help other scientists to advance their study by choosing appropriate characterization approaches, especially on the VO_2 phases related topics.

4. Suggested mechanism for stoichiometry gradient in VO_2 beams

The low-pressure CVD growth of VO_2 beams *via* the reduction of V_2O_5 is a wetting layer-assisted reaction.[9] As shown in **Supplementary Fig. 9a**, the nucleation and growth of solid crystals occur in the V_2O_5 liquid layer during the growth. With the increase of reaction temperature, the beam is gradually reduced to VO_2 and in a meantime the wetting layer is consumed due to the limited V supply that is further reduced in this reaction system due to the inhibition effect of SiO_2. Once the wetting layer cannot cover the whole beam any longer, one side of the VO_2 beam is exposed while the other side remains covered as shown in **Supplementary Fig. 9a & 9b**. It is believed that this spatially asymmetric reaction condition leads to the formation of lateral oxygen gradient in the CVD-grown VO_2 beam.

To investigate the exact role of the wetting layer in the redox reaction of VO_2, pre-grown M1 phase VO_2 beams are placed in the low-pressure CVD reaction system without the wetting layer (removed by 2 M Na_2CO_3 solution and heating up to 850 °C and keeping at this temperature for 20 min). In this reaction, 10 mg SiO_2 was mixed with 2 mg V_2O_5 (x = 0.2) to avoid the formation of new wetting layer, and to provide sufficient oxygen partial pressure. After the reaction, the VO_2 beams remain in M1 phase, implying that the wetting layer during the growth may provide the path for the oxygen injection. This discovery supports the claim that the uneven distribution of the wetting layer accounts for the laterally asymmetric stoichiometry of VO_2 beams. If the pre-grown M1 phase VO_2 beams without wetting layer are heated to 850 °C in the presence of 2 mg WO_2 at 3 Torr, the VO_2 beams will be heavily etched by W vapor as shown in **Supplementary Fig. 9c** for the heavily W-doped samples. This fact indicates that, in the absence of the wetting layer, the reaction between VO_2 and W vapor happens. These discoveries may account for the higher oxygen gradient in the W-doped VO_2 beams prepared by the SiO_2-assisted CVD growth.

In addition, the lateral oxygen gradient, i.e., the thermal-driven self-bending, is usually seen in the VO_2 beams with a large width (> 1 μm) as shown in Supplementary Movie 1 and Supplementary Movie 2. If the diameter of VO_2 beams is comparable with the thickness of the wetting layer (hundreds of nanometers), the beams may be completely covered even though the wetting layer is being consumed. As a result, the oxygen gradient is hardly produced in the slim VO_2 beams. This discovery also well supports the proposed mechanism for the asymmetric stoichiometry distribution.

5. Electrical measurements of VO_2 beams

The electrical measurements are expected to reflect the intrinsic properties of as-grown nonstoichiometric VO_2 beams. As shown in **Supplementary Fig. 10a-10c**, all the beams undergo a colossal resistivity change at 4-5 orders of magnitude across the MIT transition which is comparable or superior to the reported high-quality VO_2 single crystals prepared by vapor transport methods. It is discovered that the measured

conductivities of metallic VO_2 beams are ~2×10^4 S/m, which are slightly lower than the suggested value of the VO_2 single crystals at the order of 1×10^5 S/m. The depressed electrical conductivity may be attributed to many factors, such as the increased oxygen content in the lattice[10], the inevitable contact resistance in two-terminal devices, and the imperfect contact caused by the strain/stress of VO_2 beams across their phase transitions.

Supplementary Fig. 10a shows that there is a gradual change in the resistivity of stoichiometric VO_2 (M1) beams across the MIT upon heating, different from the first-order transition of free-standing beams observed by optical microscopy. It is noted that the clamped beam in the electrical measurement is subjected to tensile stress during the M1→R transition. This is because of the fixed length and the lattice shrinkage of VO_2, where the nucleation/growth of R domains is prevented by the stress-induced formation of M2 domains at the interfaces between R and M1 domains (**Supplementary Fig. 11**). In contrast, the resistivity-temperature curve is quite sharp across the R→M1 transition upon cooling because the nucleation of the M2/T phase is not supported under compressive stress or zero stress. The resistivity-temperature curves in **Supplementary Fig. 10b & 10c** are sharp in both heating and cooling cycles. This implies that the oxygen-rich VO_2 beams demonstrate a superior first-order MIT to the stoichiometric VO_2 crystals that are sensitive to external stress. Notably, a decrease of resistivity is discovered by a factor of ~0.8 across the M2→T transition at ~30 °C in **Supplementary Fig. 10b**; this is reported in trivalent metal-doped VO_2 crystals.[11,12] As for the undoped VO_2, the strain-stabilized M2 phase demonstrates a higher resistivity than M1 phase at similar temperature, i.e., $\rho_{M2}/\rho_{M1} \approx 2.3$, and thus, the conductivity of the intermediate T phase is better than M2 phase and poorer than M1 phase.[2,13] It is believed that the increased holes (with lower mobility than electrons) and the structural distortion result in the lower conductivity of oxygen-rich VO_2.[12] The presented electrical measurements of nonstoichiometric beams fill the gap of transport study of undoped oxygen-rich VO_2 single crystals and give solid experimental supports for previous related reports.

The hysteresis has been widely observed in the metal-insulator transition of VO_2,

resulted from the supercooling during the R→M (M1 and M2) transition. We have discussed the external stress of VO_2 beams during the electrical measurements. It is noted that the VO_2 beams are subjected to a tensile stress during heating while they are under a compressive stress upon cooling. The asymmetric stress along the VO_2 beams should cause the asymmetric phase transition kinetics: the tensile strain slightly increases the phase transition temperature while the compressive strain reduces the phase transition temperature, according to the well-investigated strain-temperature phase diagram[2,14]. In addition to the electrical measurements, we also observed the evident supercooling of free-standing VO_2 beams via optical microscopy and Raman spectroscopy, indicating that the external stress might not be the only governing factor. Fan et al.[15] reported different phase transition mechanisms between the M→R transition during heating and R→M transition during cooling by analyzing their nucleation processes. They attributed the supercooling of R→M transition in free-standing VO_2 beams to the limited concentration of point defects that would work as the nucleation site of R→M transition but not in the M→R transition. Fan et al. reported a hysteresis width of ~13 °C in a freestanding VO_2 (M) nanowire, $T_{M\rightarrow R}$ = ~69 °C and $T_{R\rightarrow M}$ = ~56 °C, very close to our results. Therefore, the large supercooling may indicate that the CVD-grown VO_2 beams have relatively few defects.

6. Characterizing W-doped VO_2 beams

The EDS mappings of V, O, and W along a W-doped M2-R type SCVA in **Supplementary Fig. 12** show that V and W are uniformly distributed in the lateral direction of M2-R SCVA, while the V and O are uniformly distributed along the axial direction of the SCVA. As expected, the W-doped VO_2 beam has an asymmetric distribution of oxygen along its lateral direction which drives the colossal bending actuation as shown in **Figure 4**. In addition, the axial gradient of W-doping is discovered in this beam (also reported by Lee et al.[16]). The temperature-dependent Raman spectra of the M2-R type SCVA (**Supplementary Fig. 13**) show that, at room temperature, the SCVA has M1 phase in its one side and, in the other side, it is occupied

by T phase. Upon heating, the M1 phase is directly converted to R phase while the other side turns to M2 phase, leading to the colossal bending as described in the main text.

It is noted that the melting point of WO_2 (1500-1600 °C) is close to that of SiO_2 (~1700 °C), but no Si doping is discovered in the VO_2 lattice. We were in a thought that SiO_2 is an inert oxide and cannot participate in the CVD growth of crystals. Therefore, we always use quartz (SiO_2) substrates, quartz boat and quartz tube to produce the VO_2 single crystals and other materials, including two-dimensional transition metal dichalcogenides, graphene, etc. It is noted that the Si-doping in these materials has been rarely reported and we would like to give some explanations about this phenomenon in the following.

Compared with the melting point, the vapor pressure of oxides is the factor that really matters in the CVD reactions. The tungsten oxides (WO_3, WO_2) are volatile above 800-900 °C, with a vapor pressure of ~0.2 Torr at 1023 °C,[17] while the vapor pressure of SiO_2 at ~1027 °C is measured as 10^{-13} Torr.[18] It is concluded that WO_2 is a volatile oxide at high temperature especially under low pressure while the volatility of SiO_2 is nearly negligible. Furthermore, we need to discuss another question whether VO_2 can be dopped by Si considering that the VO_2 beams are grown on the quartz substrates. We searched the related publications about Si-doped oxides and found that the Si-doping in metal oxides should be achieved by using highly active Si precursors, such as $Si[N(CH_3)_2]_4$ in atomic layer deposition[19] and tetraethyl orthosilicate (TEOS) in CVD.[20] By contrast, solid SiO_2 is chemically very stable so that huge energy is required to break the strong covalent Si-O bonds and seize the Si from the SiO_2 substrate. In addition, the substitutional doping of Si in VO_2 lattices seems to be thermodynamically unstable because of the large differences between SiO_2 and VO_2 in symmetry, bond type, bond length, etc. Consequently, we believe that the Si doping of VO_2 on the SiO_2 substrate may not happen at a temperature of 850 °C.

In conclusion, the nonvolatility of SiO_2 powder and the good stability of quartz substrate at 850 °C make the Si doping hard to achieve in the VO_2 lattice. This discussion can also be used to explain the absence of Si doping in other materials grown

in the presence of SiO_2.

7. Work speed and stability test of SCVAs

The work speed and stability of SCVAs are examined by laser pulses at room temperature as shown in **Supplementary Fig. 14**. Because of the limited irradiation area of the laser beam, the measured θ_{max} values of photo-thermal NW actuators are far smaller than those of actuators which are uniformly heated. The cut-off frequency (-3 dB attenuation frequency) of the M2-R SCVA is higher than 5 kHz. By contrast, the T-M2 SCVA has a relatively low work speed of ~1.8 kHz, which can be attributed to the slow rate of T→M2 transition. Both the SCVAs demonstrate ultralong lifetime of >10^7 working cycles.

In the above tests, the work speed and stability of cantilevered SCVAs were measured. It is known that the interaction between the VO_2 and substrate greatly affects the phase transition kinetics of VO_2[21,22]. We, therefore, compare the self-actuation process of an as-grown free-standing VO_2 beam on the rough quartz substrate with that of when transferred to the edge of a flatten SiO_2/Si substrate (**Supplementary Fig. 15**). The optical images at increasing temperatures indicate its consistent self-actuation processes accompanied with almost the same domain evolution behavior during the phase transition. In conclusion, the self-bending actuation of as-grown VO_2 beams is independent of the supporting substrate or their locations, and thus, the measured characteristics can also be used to describe the actuation performance of as-grown VO_2 beams on the substrates. This result also verifies that as-grown VO_2 beams are free-standing.

8. Energy efficiency calculation of SCVAs

According to our previous work,[23] the energy efficiency η can be calculated through Supplementary Eq. (1):

$$\eta = \frac{E_{out}}{E_{in}} = \frac{YLtW\varepsilon_0^2}{6LtW \cdot (\rho c \Delta T + H)} = \frac{Y\varepsilon_0^2}{6(\rho c \Delta T + H)} \quad (1)$$

where E_{in} is the input thermal energy for the heating stage and MIT of a VO_2 beam with the length of L, thickness of t, and width of W. E_{out} is the output mechanical energy generated by the SCVA. ε_0 is the strain across the phase transition, Y is the Young's modulus of VO_2 (~140 GPa), ρ is the density of VO_2 crystals (4.66 $g{\cdot}cm^{-3}$), c is the specific heat of VO_2 (~690 $J{\cdot}kg^{-1}{\cdot}K^{-1}$), ΔT is the temperature increase of VO_2 beam, and H is the latent heat of the phase transition that drives the bending. H values of different phase transitions of VO_2 are listed in **Supplementary Table 1**.

Obviously, the energy conversion efficiency of SCVA is independent of the geometry of VO_2 beams. It is only related to the actuation temperature window and the properties of phase transition that is used to drive the actuation.

For the M2-R type SCVA, the latent heat of M2-R transition is ~710 cal/mol, *i.e.*, 169 J/cm^{-3}, and the magnitude of ε_0 is 1.66%, the measured maximum value. The temperature window for actuation is estimated as 30 °C for a complete phase transition cycle. The calculated η of M2-R type SCVA is ~2.43%. Notably, Wang et al.[24] reported that η of VO_2 bimorph actuators utilizing the M2-R transition can reach to ~3.4%, but they used an estimated strain of 2%. If ε_0=2% is used for this system, the calculated η should be ~3.53%. Therefore, the reported result in this work is reliable and consistent with previous publications.

For the T-M2 type SCVA, it is difficult to confirm the latent heat of T-M2 transition and the corresponding ε_0 value. The temperature range of T-M2 transition may help to estimate the values. Pouget et al.[1] reported that $V_{0.997}Cr_{0.003}O_2$ underwent the T→M2 transition at ~55 °C with a latent heat of ~164 cal/mol (*i.e.*, 39 J/cm^3). In the present study, the T-M2 SCVA has ε_0 of ~0.63% at a similar temperature. Therefore, the calculated η of the T-M2 type SCVA is ~0.69%.

Supplementary References

1. Pouget, J. P. *et al.* Dimerization of a linear heisenberg chain in the insulating phases of $V_{1-x}Cr_xO_2$. *Phys. Rev. B* **10,** 1801–1815 (1974).
2. Park, J. H. *et al.* Measurement of a solid-state triple point at the metal insulator transition in VO_2. *Nature* **500,** 431–434 (2013).
3. Cheng, C., Liu, K., Xiang, B., Suh, J. & Wu, J. Ultra-long, free-standing, single-crystalline vanadium dioxide micro/nanowires grown by simple thermal evaporation. *Appl. Phys. Lett.* **100,** 103111 (2012).
4. Shi, R. *et al.* Oxide inhibitor-assisted growth of single-layer molybdenum dichalcogenides (MoX_2,X = S, Se, Te) with controllable molybdenum release. *ACS Nano* **14,** 7593–7601 (2020).
5. Brückner, W. Structural relations between the VO_2 phases. *Krist. und Tech.* **16,** K28–K31 (1981).
6. Chamberland, B. L. New defect vanadium dioxide phases. *J. Solid State Chem.* **384,** 377–384 (1973).
7. Zhao, C. *et al.* Simple and fast fabrication of single crystal VO_2 microtube arrays. *Commun. Mater.* **1,** 28 (2020).
8. Cheng, C. *et al.* Self-assembly and horizontal orientation growth of VO_2 nanowires. *Sci. Rep.* **4,** 5456 (2014).
9. Strelcov, E., Davydov, A. V., Lanke, U., Watts, C. & Kolmakov, A. In situ monitoring of the growth, intermediate phase transformations and templating of single crystal VO_2 nanowires and nanoplatelets. *ACS Nano* **5,** 3373–3384 (2011).
10. Griffiths, C. H. & Eastwood, H. K. Influence of stoichiometry on the metal-semiconductor transition in vanadium dioxide. *J. Appl. Phys.* **45,** 2201–2206 (1974).
11. Bruckner, W., Gerlach, U., Bruckner, H. P., Moldenhauer, W. & Oppermann, H. Influence of nonstoichiometry on the phase transitions in Ga-, Al-, and Fe-

doped VO_2. *Phys.Stat.Sol.(a)* **42,** 295–303 (1977).

12. Bruckner, W. *et al.* Phase transitions and semiconductor-metal transition in $V_{1-x}Ga_xO_2$ single crystals. *Phys.Stat.Sol.(a)* **38,** 93–102 (1976).
13. Cao, J. *et al.* Extended mapping and exploration of the vanadium dioxide stress-temperature phase diagram. *Nano Lett.* **10,** 2667–2673 (2010).
14. Cao, J. *et al.* Strain engineering and one-dimensional organization of metal–insulator domains in single-crystal vanadium dioxide beams. *Nat. Nanotechnol.* **4,** 732–737 (2009).
15. Fan, W. *et al.* Large kinetic asymmetry in the metal-insulator transition nucleated at localized and extended defects. *Phys. Rev. B* **83,** 235102 (2011).
16. Lee, S. *et al.* Axially engineered metal-insulator phase transition by graded doping VO_2 nanowires. *J. Am. Chem. Soc.* **135,** 4850–4855 (2013).
17. Millner, T. & Neugebauer, J. Volatility of the oxides of tungsten and molybdenum in the presence of water vapour. *Nature* **163,** 601–602 (1949).
18. Samsonov, G. V. *The Oxide Handbook*. (IFI/Plenum, New York, 1973).
19. Zhou, D. *et al.* Wake-up effects in Si-doped hafnium oxide ferroelectric thin films. *Appl. Phys. Lett.* **103,** 192904 (2013).
20. Potter, D. B., Powell, M. J., Darr, J. A., Parkin, I. P. & Carmalt, C. J. Transparent conducting oxide thin films of Si-doped ZnO prepared by aerosol assisted CVD. *RSC Adv.* **7,** 10806–10814 (2017).
21. Pendse, S. *et al.* Tuning phase transition kinetics via van der Waals epitaxy of single crystalline VO_2 on hexagonal-BN. *J. Cryst. Growth* **543,** 125699 (2020).
22. Wu, J. *et al.* Strain-induced self organization of metal-insulator domains in single-crystalline VO_2 nanobeams. *Nano Lett.* **6,** 2313–2317 (2006).
23. Shi, R. *et al.* Single-crystalline vanadium dioxide actuators. *Adv. Funct. Mater.* **29,** 1900527 (2019).
24. Wang, K. *et al.* Performance limits of microactuation with vanadium dioxide as a solid engine. *ACS Nano* **7,** 2266–2272 (2013).
