# Supplementary material for: Phase management in single-crystalline vanadium dioxide beams
Source: Nat Commun. 2021 Jul 9;12:4214. doi: 10.1038/s41467-021-24527-5 (PMC8270972; doi:10.1038/s41467-021-24527-5)
Supplement: Supplementary file 3 — Description of Additional Supplementary Files [file 41467_2021_24527_MOESM3_ESM.pdf]

Description of Additional Supplementary Files

File Name: Supplementary Movie 1
Description: **In-situ optical imaging of T-M2 SCVAs upon heating.** The measurement temperature range is 25-45 °C.

File Name: Supplementary Movie 2
Description: **In-situ optical imaging of M2-R SCVAs upon heating.** The measurement temperature range is 25-60 °C.

File Name: Supplementary Movie 3
Description: **In-situ optical imaging of the M2-R SCVA in Figure 4c upon heating.** The measurement temperature range is 30-60 °C.
